# Cardiovascular outcomes for Australian women with rheumatic heart disease during pregnancy: A retrospective linked data analysis, 2002–2017

**DOI:** 10.1111/aogs.70235

**Published:** 2026-05-28

**Authors:** Ingrid Stacey, Mohammed Junaid, Holger W. Unger, Geraldine Vaughan, Ye’elah Berman, Vicki Wade, Lee Nedkoff, James Marangou, Judith M. Katzenellenbogen

**Affiliations:** ^1^ Cardiovascular Epidemiology Research Centre University of Western Australia Perth Western Australia Australia; ^2^ Cardiology Population Health Laboratory Victor Chang Cardiac Research Institute Darlinghurst New South Wales Australia; ^3^ Dental School and Oral Health Centre of Western Australia University of Western Australia Nedlands Western Australia Australia; ^4^ The Kids Research Institute Australia Perth Children’s Hospital Nedlands Western Australia Australia; ^5^ Department of Obstetrics and Gynaecology Royal Darwin Hospital Tiwi Northern Territory Australia; ^6^ Menzies School of Health Research Charles Darwin University Casuarina Northern Territory Australia; ^7^ School of Health, Medical and Applied Sciences CQ University Rockhampton Queensland Australia; ^8^ Medical School, Division of Obstetrics and Gynaecology University of Western Australia Perth Western Australia Australia; ^9^ King Edward Memorial Hospital Subiaco Western Australia Australia; ^10^ Department of Cardiology Royal Perth Hospital Perth Western Australia Australia

**Keywords:** Aboriginal and Torres Strait Islander, Australia, cardiovascular disease, cohort study, linked data, pregnancy, rheumatic heart disease

## Abstract

**Introduction:**

Rheumatic heart disease (RHD) is the acquired autoimmune heart valve damage resulting from untreated infection with the *Streptococcus pyogenes* bacterium, which affects people experiencing socioeconomic disadvantage globally. This study measured RHD‐associated major adverse cardiovascular events (MACE) and the increased risk associated with pregnancy among women diagnosed with RHD.

**Material and Methods:**

Population‐level analysis of all births to women with RHD in four Australian jurisdictions was conducted, which covered 71% of the total population and 88% of the Aboriginal and Torres Strait Islander population (a group who experience some of the highest RHD rates reported globally). A retrospective cohort study using linked RHD register and midwives, hospital, and death data collections was designed. Females with at least one birth record aged 12–44 years, whose first RHD diagnosis occurred prior to 20 weeks' gestation and age < 35 years, were identified during 2002–2017. Survival methods (incorporating mixed effects and time‐varying covariates) estimated proportions and hazard ratios. Probability of hospitalization for new RHD‐associated MACE was measured for pulmonary hypertension secondary to left heart disease, heart failure, valvular surgery, stroke, infective endocarditis, atrial fibrillation, acute pulmonary edema, cardiomyopathy, and/or death.

**Results:**

We identified 558 pregnancies in women with uncomplicated RHD (345 women) and 88 pregnancies in women with complicated RHD (60 women). During pregnancy, 4.5% of women with uncomplicated RHD and 31.8% of women with complicated RHD experienced new RHD‐associated MACE. Risk of RHD‐associated MACE was three‐ to six‐fold higher during periods of pregnancy (compared with non‐pregnancy) and did not differ by RHD stage.

**Conclusions:**

After 20 weeks of gestation, women with RHD experienced RHD‐associated MACE outcomes at frequencies that were contingent upon RHD stage at 20 weeks of gestation. Awareness of RHD status before 20 weeks of gestation, especially in regions where RHD is endemic, is critical for ensuring women's cardiovascular health in pregnancy and beyond.

## Graphical abstract

This retrospective cohort study used linked data to measure the proportion of women with rheumatic heart disease (RHD) who experienced major adverse cardiovascular events (MACE) after 20 weeks of gestation. Before birth, 4.5% of women with initially uncomplicated RHD and 31.8% of women with initially complicated RHD experienced RHD‐MACE.

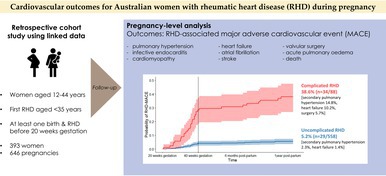

**Abbreviations**
ARF: acute rheumatic fever
CKD: chronic kidney disease
COPD: chronic obstructive pulmonary disease
ERASE: End RHD in Australia Study of Epidemiology
ICD‐10‐AM: International Statistical Classification of Diseases and Related Health Problems, Tenth Revision, Australian Modification
IHD: Ischemic heart disease
NSW: New South Wales
NT: Northern Territory
RHD: Rheumatic heart disease
SA: South Australia
WA: Western Australia
WHF: World Heart Federation

**Key message**

Evidence from this study highlights the need for clinician awareness of acute rheumatic fever and rheumatic heart disease guidelines and supports the need for specialized cardio‐obstetric services, including remote outreach, that cater to populations at high risk of rheumatic heart disease.

## 1 INTRODUCTION

Rheumatic heart disease (RHD), an acquired heart condition associated with socio‐environmental disadvantage,^1^ disproportionately affects Aboriginal and Torres Strait Islander (First Nations) people in Australia.^2^, ^3^, ^4^ The End RHD in Australia: Study of Epidemiology (ERASE) found that women contributed 67.2% of RHD cases under 55 years, with prevalence peaking during childbearing years.^2^, ^5^ RHD complications can include atrial fibrillation, heart failure, surgical intervention, endocarditis, stroke, and/or death.^3^, ^4^ Recognizing this, the 2023 World Heart Federation guidelines for the echocardiographic diagnosis of RHD (hereafter “WHF guidelines”) include staging criteria aligned with the risk of future complications.^6^

Pregnancy can exacerbate functional impairments of the heart, such as those caused by RHD, leading to RHD‐associated major adverse cardiovascular events (MACE). Plasma volume increases by approximately 40–50% during pregnancy.^7^ Consequently, a pre‐existing moderate stenotic lesion of the aortic or mitral valve may functionally become severe, and the pregnant woman may develop symptoms for the first time.^7^ Additionally, the heart rate increases during pregnancy, which shortens the diastolic filling period.^7^ This leads to higher transvalvular pressure gradients, a mechanism that is particularly relevant in mitral stenosis.^7^

Several observational studies have described RHD diagnoses during pregnancy, a likely consequence of the increased heart strain and higher contact with healthcare providers.^8^, ^9^, ^10^ A prospective study in Uganda found that >95% of pregnant women with RHD were first diagnosed during pregnancy; 39.0% experienced heart failure, and 15.4% died.^8^ The global Registry of Pregnancy and Cardiac Disease (ROPAC) study based in low‐income countries found that 75.1% of women with RHD had mitral disease before pregnancy, and 26.7% experienced heart failure during pregnancy (1.5% died).^9^, ^10^ RHD‐associated MACE during pregnancy in women with RHD has been attributed to clinical and non‐clinical factors, including late and/or severe RHD diagnosis, lack of secondary prophylaxis, low healthcare access, and socioeconomic disadvantage.^9^, ^11^

The Australasian Maternity Outcomes Surveillance System (AMOSS) study reported 23.9% of pregnant women with RHD were diagnosed during/after pregnancy, with 22.6% experiencing an adverse cardiac event during pregnancy.^12^, ^13^ Although Australian women are diagnosed with RHD earlier and experience fewer complications than their international counterparts, inequities persist. Delayed RHD diagnoses, complications, and deaths among First Nations Australians have been attributed to poorly coordinated care, communication barriers, and workforce/resourcing issues.^3^, ^14^, ^15^, ^16^ Qualitative studies have demonstrated that new and visiting staff to high‐prevalence regions and pregnant women with RHD have a limited understanding of RHD and that culturally safe patient education available within health services could be improved.^17^, ^18^, ^19^

Australian information about RHD outcomes during pregnancy is limited to AMOSS findings, which characterized a heterogeneous cohort. Our objective was to quantify absolute and relative RHD‐associated MACE risk during pregnancy for Australian women with RHD diagnosed *before* 20 weeks' gestation. This targeted information is important for family planning, clinician education, and cardio‐obstetric service provision purposes. Our study had two objectives. Firstly, we aimed to identify pregnancy‐level risk predictors of new RHD‐associated MACE after 20 weeks of gestation and up to 1 year postpartum for women with RHD. Secondly, we aimed to quantify the overall increased risk of new RHD‐associated MACE for women with RHD during periods of pregnancy relative to non‐pregnancy.

## 2 MATERIAL AND METHODS

### 2.1 Study design

This retrospective cohort study included women with RHD who became pregnant during 2002–2017 (the “study period”) in four Australian jurisdictions—Western Australia (WA), Northern Territory (NT), South Australia (SA), and New South Wales (NSW). The ERASE linked data collection was used, composed of RHD register, hospitalization, midwives' birth notification, and death records, which allowed for accurate identification and longitudinal follow‐up of RHD diagnoses and pregnancies.^2^, ^3^, ^5^

### 2.2 Data sources

#### 2.2.1 ERASE data collection

ERASE contains person‐linked health records of acute rheumatic fever (ARF) and/or RHD diagnoses that are sourced from the RHD register, hospitalization, and death records covering 70% of the general and 86% of the Australian First Nations population.^5^ The combination of multiple data sources within ERASE reduces known biases associated with under‐notification of cases to RHD registers.^20^, ^21^ RHD registers contain demographic and clinical details of ARF and RHD diagnoses, echocardiography/specialist severity assessments, and secondary prophylaxis records. Hospital records contribute administrative details of inpatient admissions, including patient demographics, coded diagnostic, surgery, and procedure information (using the *International Statistical Classification of Diseases and Related Health Problems, Tenth Revision, Australian Modification* [ICD‐10‐AM] system). Where applicable, death records capture cause and date of death.

#### 2.2.2 Midwives datasets

Legislated midwives/perinatal registries capture sociodemographic, maternal health, and perinatal information (until 24 h after birth/death/discharge/transfer, whichever soonest) for all notified births ≥20 weeks' gestation (or ≥400 grams birth weight in WA). Data collection follows methods similar to those used in Norway and Finland and is subject to regular validation and quality checks (Supplement S1).^22^ Midwives' datasets were created independently for each Australian jurisdiction at different time points (Supplement S1); study period commencement in 2002 optimized data coverage across all jurisdictions.

Midwives' datasets contain information about pregnancy and childbirth documented by birth attendants. Information includes maternal demographics, antenatal care utilization, pregnancy‐related complications and risk factors, delivery method and setting, birth event information, and neonate‐related data such as gestational age at birth, birthweight, APGAR scores, and neonatal complications.

The high efficiency of data linkage and case ascertainment attained via linkage of ERASE to midwives' data is described in Supplement S1, together with information about data quality for midwives' data.

### 2.3 Study population and sample selection

**FIGURE 1:**
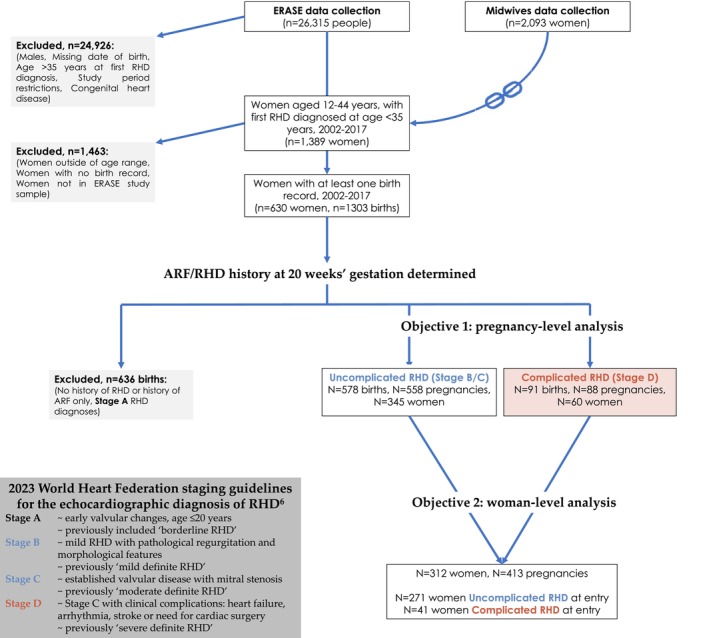
Selection of the study samples from the ERASE data collection linked to the midwives' data collection, mapped to World Heart Federation RHD staging guidelines. ARF, acute rheumatic fever; ERASE, End RHD in Australia: Study of Epidemiology; RHD, rheumatic heart disease.

Women aged 12–44 years with at least one birth record between 2002 and 2017 who were diagnosed with RHD prior to age 35 years were eligible for inclusion (Figure 1). Only women with a history of RHD before 20 weeks' gestation were selected.

RHD cases were identified from RHD register notifications and/or hospital admissions using methods previously developed within ERASE (Supplement S1), including an RHD identification algorithm that minimized over‐ascertainment bias.^21^, ^23^, ^24^ For RHD register data, the RHD diagnosis date was the clinician‐notified diagnosis date. For hospital admissions data, RHD diagnosis was the earliest date of admission to the hospital with an algorithm‐positive ICD‐10‐AM diagnosis code of RHD (I05–I09).^23^, ^25^ First RHD diagnosis was assigned where no prior hospitalization or RHD register record was available after implementing an 8‐year lookback period for hospital records.

### 2.4 Maternal RHD stage at 20 weeks' gestation

RHD stage was determined at 20 weeks' gestation based on data from ERASE. Although data were collected as per the 2012 WHF guidelines, they were subsequently mapped to the 2023 WHF guidelines.^6^ Women corresponding to RHD Stage B, C, and D RHD were identified and grouped into “uncomplicated RHD” (Stage B/C) or “complicated RHD” (Stage D) as outlined in Figure 1. Stage A RHD cases were excluded from analysis, as this stage is only relevant to ≤20‐year‐olds and was inconsistently captured by ERASE data over time and between jurisdictions. Complicated RHD was assigned when Stage D categorization was present (register records) or when RHD diagnosis coincided with heart failure, atrial fibrillation, stroke, infective endocarditis, or valvular procedure (hospital records).

### 2.5 Maternal cardiovascular outcomes

RHD‐associated MACE during and after pregnancy included hospitalization for pulmonary hypertension secondary to left heart disease, heart failure, valvular surgery, stroke, infective endocarditis, atrial fibrillation, acute pulmonary edema, cardiomyopathy, and/or death (identified using any diagnosis field, ICD‐10‐AM codes in Supplement S2). Hospitalization for new RHD‐associated MACE was measured for uncomplicated and complicated RHD at two time points: (i) during pregnancy and in the postpartum period (6 weeks after birth); and (ii) up to 1 year after birth.

### 2.6 Covariates

Demographic variables included maternal age, jurisdiction of residence, remoteness (Accessibility/Remoteness Index of Australia, ARIA+), and area‐level socioeconomic status (Index of Relative Socio‐economic Disadvantage, IRSD).^26^ Remoteness was grouped into three categories: (i) major cities/inner regional; (ii) outer regional; and (iii) remote/very remote. Socioeconomic status is similarly presented as three groups: (i) more/most disadvantaged (deciles 1–4); (ii) median disadvantaged (deciles 5–6); and (iii) less/least disadvantaged (deciles 7–10). Maternal population group was derived from multiple data sources to minimize over‐ and/or under‐ascertainment of First Nations status.^27^

Pregnancy characteristics included birth period (2002–2009 and 2010–2017, Supplement S1), RHD disease factors (e.g., register notification status, history of surgical procedures, and care provision), prior comorbidities, model of pregnancy care, parity and plurality, gestational age at birth, and delivery method. Gestational age at birth from midwives' datasets was used to impute conception (for determining pregnancy status as a time‐varying exposure) and 20 weeks' gestation dates (for RHD stage classification).

Comorbidities were identified using ICD‐10‐AM codes from hospitalization records with admission dates that preceded 20 weeks of gestation (Supplement S2). These included chronic obstructive pulmonary disease (COPD), chronic kidney disease (CKD), ischemic heart disease (IHD), diabetes mellitus, hypertension, anticoagulant use, hemorrhage, prior pregnancy‐related complications, including pre‐term birth and stillbirth, anemia, smoking, mental health disorders, and chronic alcohol use. For modeling purposes, COPD, CKD, IHD, diabetes, and hypertension were grouped into a single category called “cardiometabolic disease”; smoking, mental health disorders, and chronic alcohol use were collapsed into an indicator called “behavioral factors.”

### 2.7 Statistical analysis

Separate pregnancy‐level (Objective 1) and woman‐level (Objective 2) analyses were conducted.

Descriptive baseline statistics for the pregnancy‐level analyses were categorized by RHD stage at 20 weeks of gestation (frequencies and proportions) for all births occurring between 2002 and 2017. Univariate and multivariate mixed effects Cox regression, which adjusted for clustering of effects per woman (due to multiple pregnancies), was used to identify risk predictors of new complication onset (events). Study entry was 20 weeks' gestation. Study exit was 1 year after birth or end of data coverage.

For woman‐level analyses, women were categorized into RHD stage based on their first pregnancy during the study period, and baseline frequencies and proportions were calculated. Age‐adjusted estimation of RHD‐associated MACE risk during pregnancy was estimated using Cox regression with all pregnancies (not just first pregnancy) included as time‐varying covariates to account for the transient effects of exposure. Study entry was the latest of the first RHD diagnosis or January 1, 2010. Study exit was the earliest of December 31, 2017, or end of data coverage; events corresponded to maternal RHD‐associated MACE outcomes as described above.

### 2.8 Author positioning statement and patient involvement

The research team comprised one First Nations academic with clinical expertise in cardiac nursing (VW) and seven allies, with extensive combined experience in First Nations pregnancy and cardiovascular health care provision and research. Coauthor VW established the culturally safe patient support program “Champions4Change,” designed and led by First Nations peoples with lived experience of RHD.^28^ Consequently, the framing of this research was conducted to benefit people living with RHD.

## 3 RESULTS

We identified 646 pregnancies in women with a history of RHD at 20 weeks' gestation (Figure 1). Most pregnancies were in women with uncomplicated RHD, corresponding to 558 pregnancies among 345 women. There were 88 pregnancies among 60 women with complicated RHD, with two‐thirds of these women having had valvuloplasty, valvotomy, or valve replacement surgery prior to 20 weeks' gestation (Table 1).

**TABLE 1:** Pregnancy characteristics, stratified by RHD complication status at 20 weeks of pregnancy^a^.

		RHD status at 20 weeks (*n*, column %)^a^	
		Uncomp. (*n* = 558)	Complicated (*n* = 88)
Pregnancy and birth characteristics			
Year of neonate's birth	2002–2009	169 (30.3)	25 (28.4)
	2010–2017	389 (69.7)	63 (71.6)
Interpregnancy interval	Not applicable	345 (61.8)	48 (54.6)
	<1.5 years	32 (5.7)	5 (6.7)
	1.5 to <2 years	36 (6.5)	<5
	2 to <5 years	107 (19.2)	24 (27.3)
	≥5 years	38 (6.8)	8 (9.1)
Parity	First	121 (21.7)	22 (25.0)
	Second	115 (20.6)	19 (21.6)
	Third	89 (16.0)	15 (17.1)
	Fourth or more	89 (16.0)	12 (13.6)
	Missing data	144 (25.4)	20 (22.7)
Plurality	Singletons	543 (97.3)	86 (97.7)
	Twins or more	15 (2.7)	<5
Gestation at birth	<32 weeks	40 (7.2)	<5
	32 to <37 weeks	89 (16.0)	16 (18.2)
	37 to <42 weeks	429 (76.9)	69 (78.4)
	≥42 weeks	0	0
Birth method	Vaginal	325 (58.2)	41 (46.6)
	Forceps	11 (2.0)	6 (6.8)
	Ventouse	29 (5.2)	<5
	Planned caesarean	78 (14.0)	24 (27.3)
	Emergency caesarean	102 (18.3)	13 (14.8)
	Missing data	13 (2.3)	0
Maternal RHD care factors			
First RHD diagnosis during pregnancy		31 (5.6)	5 (5.7)
On the RHD register at 20 weeks		454 (81.4)	59 (67.1)
Receiving secondary prophylaxis prior to 20 weeks		432 (77.4)	58 (65.9)
Echocardiograph in 6 months prior to 20 weeks		84 (33.9)	11 (31.4)
Valvular repair surgery prior to 20 weeks^b^		–	52 (59.1)
Valvular replacement surgery prior to 20 weeks^b^		–	11 (12.5)
Maternal obstetric care factors			
Timing of first antenatal visit	Before 20 weeks of gestation	365 (65.4)	60 (68.2)
	20–29 weeks of gestation	100 (17.9)	14 (15.9)
	30 weeks of gestation and later	31 (5.6)	9 (10.2)
	Not recorded	62 (11.1)	5 (5.7)
Birth setting	Hospital	402 (72.0)	68 (77.3)
	Birth or community health center	11 (2.0)	<5
	Other	5 (0.9)	<5
	Not recorded	140 (25.1)	18 (20.5)
Birth attendant	Obstetrician	45 (8.1)	5 (5.7)
	Medical practitioner	42 (7.5)	10 (11.4)
	Midwife	141 (25.3)	18 (20.5)
	Other (mixed care, high‐risk units)	176 (31.5)	26 (29.5)
	Not recorded	154 (27.6)	29 (33.0)
Comorbidities and prior complications^c^			
Complicated previous pregnancy		306 (54.8)	61 (69.3)
Anticoagulants		18 (3.2)	7 (8.0)
Ischemic heart disease		11 (2.0)	5 (5.7)
Chronic obstructive pulmonary disease		30 (5.4)	7 (8.0)
Chronic kidney disease		28 (5.0)	<5
Hypertension		19 (3.4)	<5
Anemia		85 (15.2)	27 (30.7)
Chronic alcohol use		49 (8.8)	10 (11.4)
Mental health		12 (2.2)	<5
Smoking		214 (38.4)	39 (44.3)
Diabetes		41 (7.4)	9 (10.3)

Abbreviations: *n*, number; RHD, rheumatic heart disease; Uncomp., uncomplicated.

^a^ This is a pregnancy‐based analysis, showing characteristics at 20 weeks of gestation. Individual women could contribute multiple times.

^b^ Total of all valvuloplasty, valvotomy, or valve replacement surgeries was *n* = 58 (65.9%); some individuals had a history of both.

^c^ Hemorrhage not reported, since <5 admissions in each group.

### 3.1 Pregnancy‐level characteristics

Most pregnancies occurred in the latter half of the study period and were singleton births delivered at full term (Table 1). Among pregnancies with initially uncomplicated RHD, 21.7% were to primigravida mothers. For pregnancies on a background of complicated RHD, 25.0% were among primigravidae. Pregnancies with initially uncomplicated RHD resulted in proportionally fewer planned caesarean births (*n* = 78/558, 14.0%) than complicated RHD pregnancies (*n* = 24/88, 27.3%).

Few first‐ever RHD diagnoses were made during the first 20 weeks of pregnancy (5.6% uncomplicated RHD, 5.7% complicated RHD, Table 1). Most RHD cases had been notified to the register prior to 20 weeks' gestation and/or had been prescribed secondary prophylaxis for management of RHD (Table 1). Approximately one‐third of pregnancies (any stage) had received an echocardiogram within 6 months of reaching 20 weeks' gestation, and two‐thirds of pregnancies with complicated RHD also had a history of valvular procedure (Table 1).

Although two‐thirds of pregnancies had their first antenatal visit before 20 weeks' gestation (Uncomplicated RHD, 65.4%; complicated RHD, 68.2%, Table 1), 17.9% (*n* = 100) of uncomplicated RHD and 15.9% (*n* = 14) of complicated RHD pregnancies did not attend a maternal care provider until 20–29 weeks' gestation. Hospitals were the setting of >70% of births to women with RHD, and birth attendants were heterogeneous, comprising clinicians, midwives, and multidisciplinary teams (Table 1). Missing data for birth setting, birth attendant, and parity were frequent in this study sample (Table 1); these variables were not included in subsequent models due to missingness.

Comorbidities and risk factors most frequently recorded among pregnancies on a background of uncomplicated RHD were complicated previous pregnancy (54.8%), history of smoking (38.4%), and anemia (15.2%). Comorbidity patterns were similar for pregnancies with complicated RHD, who proportionally had more frequent complicated previous pregnancy (69.3%), smoking (44.3%), and anemia (30.7%).

### 3.2 Woman‐level characteristics

When considering just the index (first) pregnancy (Objective 2), investigating woman‐level characteristics, most women had uncomplicated RHD at their index pregnancy by 20 weeks' gestation (*n* = 345 women, *n* = 558 pregnancies). Complicated RHD at 20 weeks' gestation was experienced by 15.3% of the study sample (*n* = 60 women, *n* = 88 pregnancies). For both uncomplicated and complicated RHD groups, women were predominantly remote‐residing, highly disadvantaged, and First Nations; the complicated RHD study group comprised a higher proportion of migrant women, however (Table 2).

**TABLE 2:** Baseline demographic features of women with RHD who became pregnant during the study, stratified by RHD complication status at 20 weeks of index pregnancy during the study period.

	RHD status at first recorded pregnancy^a^ (*n*, column %)	
	Uncomplicated RHD (*n* = 345)^a^	Complicated RHD (*n* = 48)
Maternal age in years		
<20 years	101 (29.3)	14 (29.2)
20–29 years	186 (53.9)	21 (43.8)
30–44 years	58 (16.8)	13 (27.1)
Maternal population group		
First Nations	331 (95.9)	39 (81.3)
Born in low‐/middle‐income country	9 (2.6)	8 (16.7)
Other Australian	5 (1.5)	<5
Year of study entry (RHD diagnosis)		
2002–2009	248 (71.9)	32 (66.7)
2010–2017	97 (28.1)	16 (33.3)
Jurisdiction of residence		
Northern Territory/South Australia^b^	245 (71.0)	29 (60.4)
Western Australia	86 (24.9)	8 (16.7)
New South Wales	14 (4.1)	11 (22.9)
Geographical remoteness		
Major cities/inner regional	15 (4.4)	12 (25.0)
Outer regional	<5	<5
Remote/very remote	326 (94.5)	35 (72.9)
Index of socioeconomic disadvantage		
More/most disadvantaged	322 (93.3)	43 (89.6)
Median disadvantage	11 (3.2)	<5
Less/least disadvantaged	9 (2.6)	<5
Unknown	<5	0

Abbreviation: RHD, rheumatic heart disease.

^a^ *n* = 12 women with uncomplicated pregnancies had a subsequent complicated pregnancy during the study period; these were assigned to the uncomplicated category at baseline.

^b^ Records from Northern Territory and South Australia were linked cross‐jurisdictionally, so they are presented as an aggregate.

### 3.3 Cardiovascular outcomes

**FIGURE 2:**
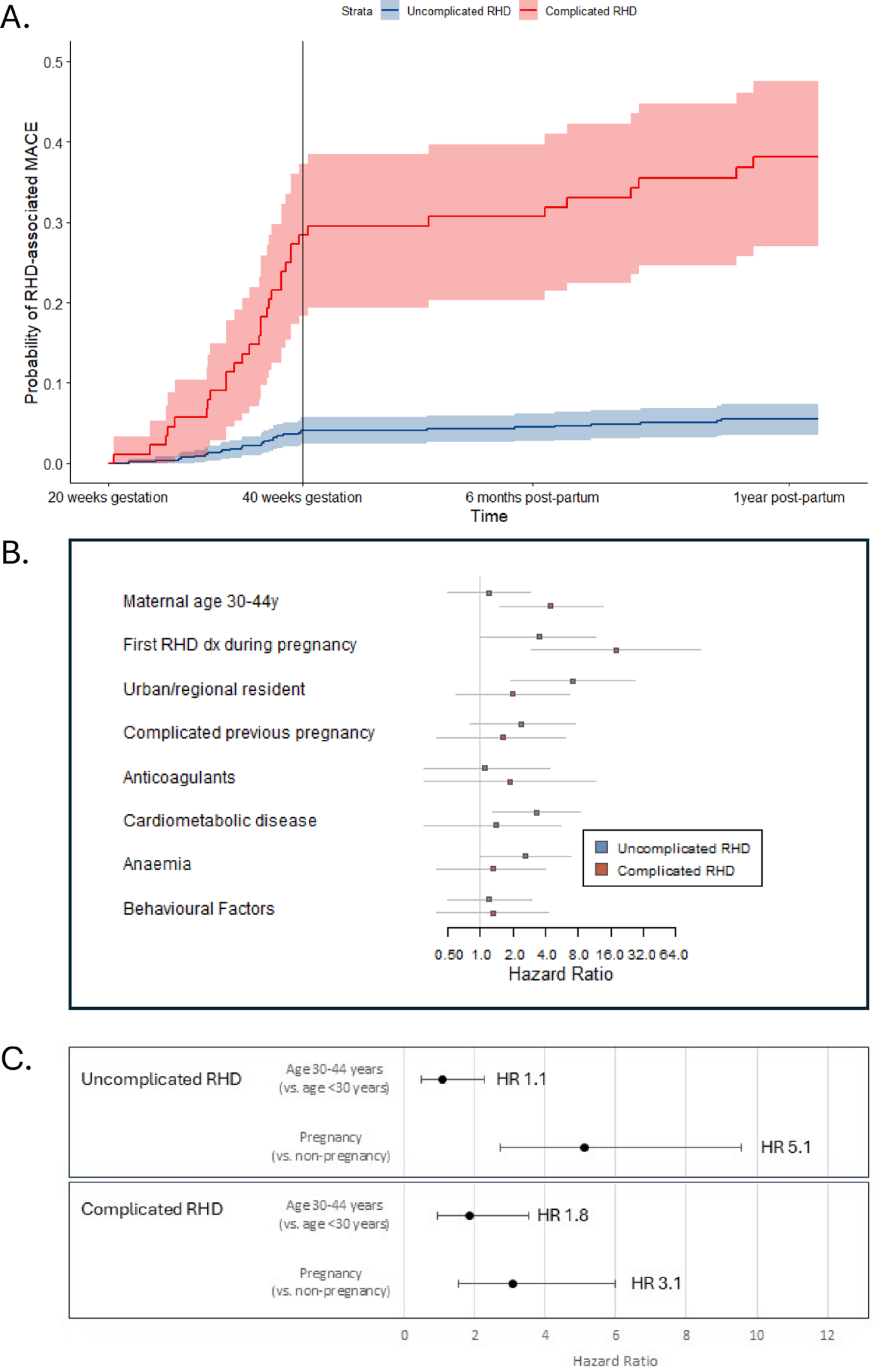
(A) Kaplan–Meier of RHD‐associated MACE onset from 20 weeks of gestation until 1‐year postpartum. (B) Risk predictors of RHD‐associated MACE during singleton pregnancies and up to 1 year after birth, stratified by RHD complication status at 20 weeks of pregnancy. Behavioral factors are an aggregation of mental health conditions, smoking, and/or chronic alcohol use. (C) RHD‐associated MACE risk during the study period by age and pregnancy status (pregnant vs. non‐pregnant, time varying), stratified by baseline RHD complication status at first pregnancy. Dx, diagnosis; HR, hazard ratio; MACE, major adverse cardiovascular event; RHD, rheumatic heart disease.

During pregnancy and up to 6 weeks after birth, 4.5% (*n* = 25) of women with uncomplicated RHD experienced a non‐fatal complication, and 31.8% (*n* = 28) of women with complicated RHD experienced a new non‐fatal complication (Figure 2A, Supplement S3). Less than five women died during pregnancy or during the postpartum period in both groups (Supplement S3). The most frequently experienced outcomes during pregnancy‐level follow‐up were secondary pulmonary hypertension (2.2%, *n* = 12 uncomplicated; 13.6%, *n* = 12 complicated, Supplement S3) and heart failure (1.4%, uncomplicated RHD; 9.1%, *n* = 8 complicated RHD, Supplement S3).

### 3.4 Objective 1—risk predictors of cardiovascular complication during pregnancy (pregnancy‐level analyses)

Univariate risk predictors of RHD‐associated MACE after 20 weeks' gestation and up to 1 year after birth included maternal age ≥ 30, first‐ever RHD diagnosis during pregnancy, urban/regional residence, and having a hospital‐recorded comorbidity prior to pregnancy (Supplement S4). Multivariate analyses revealed that among women with complicated RHD, maternal age 30–44 years at 20 weeks of gestation was associated with a 4.4‐fold higher risk of RHD‐associated MACE later in the same pregnancy compared with women aged under 30 years (95% confidence interval [CI]: 1.5–13.4, Figure 2B, Supplement S4). Having a first‐ever diagnosis of uncomplicated RHD during a given pregnancy was associated with a 3.5‐fold higher risk of RHD‐associated MACE in later pregnancy and up to 1‐year postpartum (95% CI: 1.0–11.7); this risk was 18.0‐fold higher for women diagnosed with first‐ever complicated RHD in pregnancy (95% CI: 3.0–109.0, Figure 2B, Supplement S4). Among women with uncomplicated RHD, living in an urban/regional area was associated with a 7.2‐fold higher RHD‐associated MACE risk than remote or very remote residing women (Figure 2B, Supplement S4). Having cardiometabolic disease (hazard ratio [HR] = 3.3, 95% CI: 1.3–8.4) or anemia (HR = 2.6, 95% CI: 1.0–6.9) prior to 20 weeks gestation in women with uncomplicated RHD was associated with increased RHD‐associated MACE risk after 20 weeks gestation during the same pregnancy, compared with those without these comorbidities (Figure 2B, Supplement S4).

### 3.5 Objective 2—pregnancy‐associated risk of cardiovascular complication (woman‐level analysis)

The subset of women with RHD who experienced pregnancy/pregnancies during 2010–2017 had similar baseline characteristics to the Objective 1 cohort (*n* = 312 women, *n* = 413 pregnancies, Supplement S5). The age‐adjusted hazard of RHD‐associated complications among women with uncomplicated RHD was 5.11 times higher (95% CI: 2.73–9.55) during periods of pregnancy than non‐pregnancy (Figure 2C, where pregnancy was the time from conception until 6 weeks postpartum and all pregnancies per woman were considered). Among women with complicated RHD, the age‐adjusted risk of new RHD‐associated complication onset during pregnancy was 3.05 times higher (95% CI: 1.55–5.99) during pregnancy and not different from women with uncomplicated RHD (Figure 2C).

## 4 DISCUSSION

This study is the first to quantify the elevated risk of RHD‐associated MACE during pregnancy and up to 1 year after birth among a cohort of women with RHD in both relative and absolute terms. Since most pregnant women with RHD in Australia are First Nations, our findings are most generalizable to First Nations women. Women with RHD experienced 3 to 6 times higher risk of RHD‐associated MACE during pregnancy relative to periods of non‐pregnancy, regardless of RHD complication status at 20 weeks' gestation. In absolute terms, the risk of RHD‐associated MACE during pregnancy and up to 1 year postpartum was 5.2% for women with uncomplicated RHD at 20 weeks' gestation and 38.6% for women with complicated RHD at 20 weeks' gestation, with secondary pulmonary hypertension and heart failure events during late pregnancy and immediately postpartum most frequent. First‐ever diagnosis of RHD during the first 20 weeks of pregnancy was strongly associated with subsequent new RHD‐associated MACE in pregnancy and during the 1‐year postpartum period. These findings support the need for coordinated interdisciplinary care, incorporating culturally safe cardiac, obstetric, and primary services, to support women with diagnosed RHD who are experiencing or planning pregnancy.

RHD‐associated MACE measured among women with complicated RHD were similar in our Australian study to measurements reported internationally. The ROPAC, based mostly on women from emerging economies, reported that 15.8% of women with RHD experienced heart failure during pregnancy, which was comparable to our complicated RHD group (14.8%).^10^ Complication rates among women with RHD who became pregnant in our Australian study are similar to those observed in middle‐income countries such as Fiji^29^ and lower than those in low‐income countries such as Uganda when compared internationally.^8^, ^10^, ^29^ Most women had uncomplicated RHD at 20 weeks' gestation, with only 1.4% progressing to heart failure—a likely consequence of better access to health care resources in Australia when compared with women residing in countries with emerging economies.

Our findings support conclusions from international and Australian studies that timely RHD diagnosis and management as part of family planning in high‐risk populations has the potential to prevent pregnancy‐associated RHD‐associated MACE among women with RHD.^10^, ^15^, ^17^, ^19^, ^30^ At the individual pregnancy level, first‐ever RHD diagnosis in early pregnancy was strongly predictive of RHD‐associated MACE after 20 weeks' gestation for women with either uncomplicated or complicated RHD (Figure 2). Among women with uncomplicated RHD, urban or regional residence was associated with RHD‐associated MACE risk, which we have hypothesized is due to delayed RHD diagnosis and lower clinician awareness of ARF/RHD than in remote areas.^3^ However, providing timely obstetric and cardiac healthcare to our cohort was challenging, as evidenced by <70% of women receiving antenatal care prior to 20 weeks' gestation, compared with 88.7% in the general Australian population in 2017.^31^ Additionally, only one‐third of women had evidence of a recent echocardiogram, despite most women being notified to RHD registers and receiving secondary prophylaxis. Barriers and enablers to healthcare services can include physical distance, coordination of care, trust, and cultural safety.^32^ Enhancing healthcare access for women of childbearing age in areas with endemic RHD is critical. Examples include non‐expert screening for RHD in low‐resource settings to increase echocardiogram access and health promotion campaigns such as “*See, Stop, Scan*” that encourage healthcare engagement during early pregnancy.^30^, ^33^, ^34^, ^35^

Our findings highlight the importance of clinician awareness about the 2023 WHF guidelines and relevant local/national ARF/RHD guidelines to inform women about the risk of cardiovascular complications during pregnancy, which are relevant to community, primary care, and hospital health care workers. Specifically, women with complicated RHD must be informed of their heightened age and pregnancy‐associated risk of RHD‐associated MACE, with family planning discussed as appropriate. Ideally, First Nations or migrant women who are planning pregnancy or are in the early stages of pregnancy require an echocardiogram to identify undiagnosed RHD or other cardiac conditions, due to the high prevalence of cardiovascular disease and subsequent increased risk of heart failure.^10^, ^36^ Our findings support the need for increased awareness of RHD among maternity care providers, multidisciplinary models of obstetric care, and early monitoring for RHD in pregnancy, outlined by Australian guidelines and other research studies.^15^, ^35^, ^37^ Additionally, birthing models and policies catering for women with RHD must consider the elevated risks associated with the complicated disease.

The present study has provided the most comprehensive longitudinal analysis of women with RHD in Australia, made possible by the linkage of midwives' birth notification records to RHD diagnoses identified from multiple sources. Some limitations are inherent to this study, with data items missing or inconsistently recorded from midwives' data collections (e.g., no data on anticoagulation, inconsistent inclusion of obesity metrics, >20% records missing parity, birth setting, and birth attendant information). It is likely that pregnancy impacts have been underestimated in our study, as our births were limited to women with a midwives record; thus, women who did not receive antenatal care (likely a group at high risk) were systematically excluded from this study. Measurement of comorbidities such as hypertension and diabetes is likely under‐recorded in hospitalization data, since these are managed in primary care settings. Another potential source of bias is only including pregnancies after 20 weeks' gestation, which excludes women who experienced miscarriage or terminations (~7% of pregnancies in AMOSS).^15^ Forthcoming analysis will determine the effect of maternal cardiac complications during pregnancy on infants born to mothers with RHD.

## 5 CONCLUSION

Pregnancy substantially increases the risk of RHD‐associated MACE for First Nations women with RHD, irrespective of disease stage. Although the proportion of women with uncomplicated RHD experiencing RHD‐associated MACE is low (<5%), almost one‐third of women with complicated RHD experienced RHD‐associated MACE during pregnancy or the puerperium. This novel outcomes‐focused information allows women with RHD to make risk‐informed decisions about pregnancy according to their disease stage. It highlights the need for clinician education based on local ARF/RHD guidelines to guide patient care and supports the need for specialized cardio‐obstetric services, including remote outreach, that cater to populations at high risk of RHD.

## AUTHOR CONTRIBUTIONS

JMK is responsible for the overall content as guarantor. IS, MJ, HU, GV, YB, VW, LN, JM, and JMK contributed to the study design. IS and MJ performed data analyses with subject matter guidance from HU (obstetric), GV (maternal health/public health), YB (statistical/obstetric), VW (clinical/cultural), LN (epidemiology), JM (cardiac/public health), and JMK (statistical/public health). IS drafted the manuscript, including tables and figures. All coauthors interpreted results, generated content for discussion, critically reviewed the manuscript, and contributed to revisions.

## FUNDING INFORMATION

This project was funded by the National Health and Medical Research Council (#1146525), which incorporates external peer review (including consumer review) for scientific quality and priority assessment. LN is supported by the National Heart Foundation Future Leader Fellowship (#108106). IS is supported by the National Heart Foundation Postdoctoral Fellowship (#110335). The funding bodies did not contribute to research or manuscript writing.

## CONFLICT OF INTEREST STATEMENT

All authors declare they have no conflicts to disclose.

## ETHICS STATEMENT

Aboriginal Ethics Committees approved ERASE in WA (No. 717, approved 13 October 2016), SA (No. 04–16‐700, approved 7 December 2016), and NSW (No. 1363/18, approved 5 March 2018). Human Research Ethics Committee approvals were also obtained from Menzies School of Health Research (No. 2016–2705, approved 21 December 2016), WA Health (No. 2016/29, approved 1 July 2016), SA Health (No. HREC/16/SAH/120, approved 13 December 2016), and NSW Health (No. HREC/18/CIPHS/15, approved 22 May 2018).

## Supporting information


Table S1.
