# Supplementary material for: Cardiovascular outcomes for Australian women with rheumatic heart disease during pregnancy: A retrospective linked data analysis, 2002–2017
Source: Acta Obstet Gynecol Scand. 2026 May 28;105(8):1531–41. doi: 10.1111/aogs.70235 (PMC13356470; doi:10.1111/aogs.70235)
Supplement: Supplementary file 1 — Table S1. [file AOGS-105-1531-s001.docx]

# SUPPLEMENTAL FILES:

## Supplement 1. Technical notes on data linkage process, data source used to identify women in this study and data quality and validation of midwives data.

### Data linkage process

Person-level probabilistic linkage, both within and between source datasets, was done by jurisdictional data linkage units and supplied to ERASE investigators. The quality of these linked health data have been estimated to be very high; with one unit having reported the level of false positives (mismatches) and the level of false negatives (missed matches) both at 0.11%.(Holman et al, 1999) Probabilistic data linkage was used which was based on methods that were initially established here in WA and replicated in other jurisdictions.(Smith and Flack, 2021) Approximately 96-98% of Aboriginal children during 2005-2020 are born in obstetric units, under midwife care and will therefore have a unique medical record number which can be used preferentially in the linkage process. Linkages above the probabilistic threshold are automatically accepted (this is over 95% of records), the remainder are then subject to clerical review to ensure accurate matching by the linkage staff. Regular audits are undertaken, which includes examining Aboriginal populations, to ensure that linkage processes are accurate.

Data was supplied to ERASE researchers as de-identified records with unique person level identification numbers that facilitated merging between datasets and over time. Interstate linkage was available between SA and NT only.

The ERASE project was registered as an observational study with the Australian New Zealand Clinical Trials Registry (ACTRN12620000981921).

### Data sources for identification of women

Most women in this study had register records available, which involved echocardiographic confirmation of RHD. Additionally, the proportion of cases in ERASE with at least one register record (i.e. “register confirmation” involving echocardiography, green and red shading in the image below) has increased over the study period, meaning that the “hospital-identified” cases have decreased. ERASE investigators have conducted two detailed studies that have specifically examined how different data sources may lead to over or under ascertainment bias of RHD, and developed an algorithm for reducing this bias in hospitalisation records.(Bond-Smith et al, 2020; Thandrayen et al. 2024)


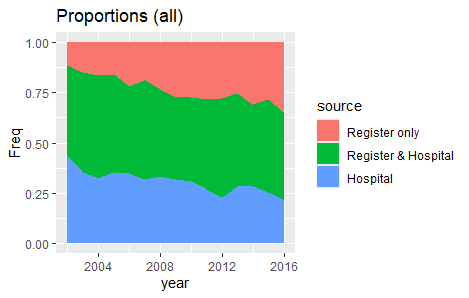


### Data quality and validation of midwives data:

Birth notification is mandatory in Australia and uses similar data collection methods to birth registries internationally, such as Norway and Finland (Lain et al, 2012). Notification forms are checked by jurisdictional health departments for omissions and possible errors. When necessary, the information is verified with the person lodging the report prior to data entry. Checking procedures are additionally performed via inbuilt mechanisms in the computerised database and validation studies are frequently undertaken and published (Downey 2007; Taylor et al, 2005; Hadfield et al, 2008 and Flood et al, 2011). The pregnancy and delivery variables included in the present study are generally >95% complete and correct.

**Temporal coverage and name of midwives datasets by jurisdiction:**

| ***Jurisdiction*** | ***Year of establishment*** | ***Midwives dataset name*** |
| --- | --- | --- |
| Western Australia (WA) | 1980 | *WA Perinatal Data Collection* |
| Northern Territory (NT) | 1986 | *NT Perinatal Registry* |
| South Australia (SA) | 2007 | *SA Perinatal Statistics Collection* |
| New South Wales (NSW) | 2001 | *NSW Perinatal Data Collection*. |

### Supplement 1 References

Bond-Smith D, Seth R, De Klerk N, Nedkoff L, Anderson M, Hung J, Cannon J, Griffiths K, Katzenellenbogen J. Development and Evaluation of a Prediction Model for Ascertaining Rheumatic Heart Disease Status in Administrative Data. *Clin Epidemiol*. 2020;12:1-14.

Downey, F. (2007). A validation study of the Western Australian Midwives’ Notification System. 2005 data. Perth: Department of Health, Western Australia. <https://www.health.wa.gov.au/~/media/Files/Corporate/general-documents/Data-collection/PDF/Midwives_Validation_Study_2007.pdf>

Flood MM, McDonald SJ, Pollock WE, Davey M-A. Data accuracy in the Victorian Perinatal Data Collection: Results of a validation study of 2011 data. *Health Information Management Journal*. 2017;46(3):113-126. doi:[10.1177/1833358316689688](https://doi.org/10.1177/1833358316689688)

Hadfield RM, Lain SJ, Cameron CA, Bell JC, Morris JM, Roberts CL.The prevalence of maternal medical conditions during pregnancy and a validation of their reporting in hospital discharge data. *Aust NZ J Obstet Gynaecol*. 2008;48:78-82.

Holman CD, Bass AJ, Rouse IL, Hobbs MS. Population-based linkage of health records in Western Australia: development of a health services research linked database. *Aust N Z J Public Health*. 1999;23(5):453-9.

Lain SJ, Hadfield RM, Raynes-Greenow CH, et al. Quality of data in perinatal population health databases: a systematic review. *Med Care*. 2012;50:e7-e20.

Smith M, Flack F. Data Linkage in Australia: The First 50 Years. *Int J Environ Res Public Health*. 2021;18(21).

Taylor LK, Travis S, Pym M. Olive E, Henderson-Smart DJ. How useful are hospital morbidity data for monitoring conditions occurring in the perinatal period? *ANZ J Obstet Gynaecol* 2005;45(1):36-4 <https://www.cherel.org.au/media/13588/validation-studies-april-2009.pdf>

Thandrayen J, Stacey I, Oliver J, Francia C, Katzenellenbogen JM, Wyber R. Estimating the true number of people with acute rheumatic fever and rheumatic heart disease from two data sources using capture-recapture methodology. *Aust Health Rev*. 2024;49.

## Supplement 2. ICD-10AM cardiovascular complications and comorbidities used in this study: (A) cardiovascular outcomes, (B) RHD-related surgical procedures and (C) comorbidities.

### Rheumatic heart disease major adverse cardiovascular events (RHD-associated MACE):

| **Study category** | **ICD-10-AM diagnosis codes and description** |
| --- | --- |
| Heart Failure | I50 Heart failure  I11.0 Hypertensive heart disease with (congestive)  heart failure  I13.0 Hypertensive heart and kidney disease with (congestive) heart failure  I13.2 Hypertensive heart and kidney disease with both (congestive) heart failure and kidney failure |
| Atrial Fibrillation | I48 Atrial fibrillation and flutter |
| Stroke | I60 Subarachnoid haemorrhage  I61 Intracerebral haemorrhage  I62 Other nontraumatic intracranial haemorrhage  I63 Cerebral infarction  I64 Stroke, not specified as haemorrhage or infarction |
| Endocarditis | I33 Acute and subacute endocarditis |
| Cardiomyopathy | I43.8 Cardiomyopathy in other diseases classified elsewhere  O90.3 Cardiomyopathy in the puerperium  I42.0 Dilated cardiomyopathy  I42.8 Other cardiomyopathies  I42.9 Cardiomyopathy, unspecified  I43.8 Cardiomyopathy in other diseases classified elsewhere  I25.5 Ischaemic cardiomyopathy |
| Secondary Hypertension | I27.2 Other secondary pulmonary hypertension |
| Acute Pulmonary oedema | J81 Pulmonary oedema |
| Cardiogenic Shock | R57 Shock, not elsewhere classified |

| RHD-related surgery procedure codes, and their major classification: | |  |
| --- | --- | --- |
| **ICD-10-AM procedure code** | **Procedure description** | **Major classification** |
| 38456-10 38483-00 38480-00 38481-00 | Open valvotomy of aortic valve  Decalcification of aortic valve leaflet  Repair of aortic valve, 1 leaflet  Repair of aortic valve, ≥2 leaflets | Repair |
| 38488-00 38488-01 38489-00 38489-01 | Replacement of aortic valve with mechanical prosthesis  Replacement of aortic valve with bioprosthesis  Replacement of aortic valve with homograft  Replacement of aortic valve with unstented homograft | Replacement |
| 38456-15  38653-04  38487-00 38485-01 38480-01 38481-01 38475-00 38477-00 | Other intrathoracic procedures on aortic valve without cardiopulmonary bypass  Other intrathoracic procedures on aortic valve with cardiopulmonary bypass  Open valvotomy of mitral valve  Decalcification of mitral valve  Repair of mitral valve, 1 leaflet  Repair of mitral valve, ≥2 leaflets  Mitral valve annuloplasty  Mitral valve annuloplasty with ring insertion | Repair |
| 38488-02 38488-03 38489-02 | Replacement of mitral valve with mechanical prosthesis  Replacement of mitral valve with bioprosthesis  Replacement of mitral valve with homograft | Replacement |
| 38485-00 38456-16  38653-05  38456-11 38480-02 38481-02 38475-01 38477-01 | Reconstruction of mitral valve annulus  Other intrathoracic procedures on mitral valve without cardiopulmonary bypass  Other intrathoracic procedures on mitral valve with cardiopulmonary bypass  Open valvotomy of tricuspid valve  Repair of tricuspid valve, 1 leaflet  Repair of tricuspid valve, ≥2 leaflets  Tricuspid valve annuloplasty  Tricuspid valve annuloplasty with ring insertion | Repair |
| 38488-04 38488-05 38489-03 | Replacement of tricuspid valve with mechanical prosthesis  Replacement of tricuspid valve with bioprosthesis  Replacement of tricuspid valve with homograft | Replacement |
| 38456-17  38653-06  38456-01 | Other intrathoracic procedures on tricuspid valve without cardiopulmonary bypass  Other intrathoracic procedures on tricuspid valve with cardiopulmonary bypass  Open valvotomy of pulmonary valve | Repair |
| 38488-06 38488-07 38489-04  38489-05 | Replacement of pulmonary valve with mechanical prosthesis  Replacement of pulmonary valve with bioprosthesis  Replacement of pulmonary valve with homograft  Replacement of pulmonary valve with unstented heterograft | Replacement |
| 38456-18  38653-07 38475-02 38477-02  38270-01 38270-02 38270-03 96222-00 | Other intrathoracic procedures on pulmonary valve w/o cardiopulmonary bypass  Other intrathoracic procedures on pulmonary valve with cardiopulmonary bypass  Aortic valve annuloplasty  Aortic valve annuloplasty with ring insertion  Percutaneous balloon aortic valvuloplasty  Percutaneous balloon mitral valvuloplasty  Percutaneous balloon pulmonary valvuloplasty  Percutaneous balloon mitral valvuloplasty using closure device | Repair |
| 38488-08 38488-09 38488-10 38488-11 | Percutaneous replacement of aortic valve with bioprosthesis  Percutaneous replacement of mitral valve with bioprosthesis  Percutaneous replacement of tricuspid valve with bioprosthesis  Percutaneous replacement of pulmonary valve with bioprosthesis | Replacement |

### Comorbidities:

| **Study category** | **ICD-10-AM diagnosis codes and description** |
| --- | --- |
| Chronic obstructive pulmonary disease | J40-J47 |
| Chronic kidney disease | I12-I13, N00-N08, N11-N16, N18, N19, N25-N28, Z49, E10.2, E11.2, E12.2, E13.2, E14.2, I15.0, I15.1, N39.1, N39.2, T82.4,  Z94.0 |
| Ischaemic heart disease | I20-I25 |
| Diabetes mellitus | E10-E14 |
| Hypertension | I10-I15 |
| Anticoagulant use | D62, D68.3 |
| Haemorrhage | I60-I62, R58 |
| Prior pregnancy-related complications, including pre-term birth and stillbirth | O10 - O16, O24, O60, O99, Z37.1 |
| Anaemia | D50-D53, D59, D62-D64 |
| Smoking | Z86.43, F17.1, F17.2, Z72.0 |
| Mental health disorders | F40, F41, F34, F38, F39, F32.0, F32.1, F32.2, F32.8, F32.9, F33.0, F33.2, F33.4, F33.5, F33.6, F33.7, F33.8, F33.9 |
| Chronic alcohol use | F10, K70, E24.4, G31.2, G62.1, G72.1, I42.6, K29.2, K85.2, K86.0, O35.4, R78.0, T51.0, T51.1, T51.9, Z50.2, Z71.4, Z72.1, Z86.41 |

## Supplement 3. RHD-associated MACE during pregnancy and up to one year after birth, stratified by RHD complication status at 20 weeks of pregnancy (lower panel)

|  | During follow up (pregnancy until one year after birth) | | During pregnancy and post-partum* | | Up to one year after birth | |
| --- | --- | --- | --- | --- | --- | --- |
|  | N | % | N | % | N | % |
| **Uncomplicated RHD (n=558)** |  |  |  |  |  |  |
| Any non-fatal complication^†^ | 29 | 5.2% | 25 | 4.5% | <5 |  |
| Secondary pulmonary hypertension | 13 | 2.3% | 12 | 2.2% | <5 |  |
| Heart failure | 8 | 1.4% | 8 | 1.4% | 0 | 0% |
| Surgery | <5 |  | <5 |  | <5 |  |
| Other^‡^ | <5 |  | <5 |  | <5 |  |
| Death | <5 |  | 0 | 0% | <5 |  |
| **Complicated RHD (n=88)** |  |  |  |  |  |  |
| Any non-fatal complication^†^ | 34 | 38.6% | 28 | 31.8% | 6 | 6.8% |
| Secondary pulmonary hypertension | 13 | 14.8% | 12 | 13.6% | <5 |  |
| Heart failure | 9 | 10.2% | 8 | 9.1% | <5 |  |
| Surgery | 5 | 5.7% | <5 |  | <5 |  |
| Other^‡^ | 7 | 8.0% | <5 |  | <5 |  |
| Death | <5 |  | 0 | 0% | <5 |  |

* up to 6 weeks after birth.

† secondary pulmonary hypertension, heart failure, stroke, infective endocarditis, atrial fibrillation, acute pulmonary oedema

‡ stroke, infective endocarditis, atrial fibrillation, acute pulmonary oedema

## Supplement 4: Risk predictors of RHD-associated MACE during singleton pregnancies and up to one year after birth, stratified by RHD complication status at 20 weeks of pregnancy.

|  |  | **Univariate analysis** | |  | **Multivariate analysis^§^** | |
| --- | --- | --- | --- | --- | --- | --- |
| **Variable*** |  | **Uncomplicated RHD n=558** | **Complicated RHD n=88** |  | **Uncomplicated RHD** | **Complicated RHD** |
| Maternal age in years | <30 years | 1 | 1 |  | 1 | 1 |
|  | 30-44 years | 2.0 (0.9-4.5) | **3.4 (1.5-7.8)** |  | 1.2 (0.5-2.9) | **4.4 (1.5-13.4)** |
| Timing of first RHD diagnosis | Non-pregnant | 1 | 1 |  | 1 | **1** |
|  | During pregnancy | 2.7 (0.9-8.1) | **11.2 (2.7-47.0)** |  | **3.5 (1.0-11.7)** | **18.0 (3.0-109.0)** |
| Population group | Non-Indigenous | 1 | 1 |  |  |  |
|  | First Nations | 0.4 (0.1-1.8) | 0.6 (0.2-1.6) |  |  |  |
| Most disadvantaged quintile |  | 1.8 (0.6-5.0) | 1.3 (0.6-3.1) |  |  |  |
| Location of residence | Urban or regional | **3.4 (1.1-10.9)** | 2.1 (0.9-4.6) |  | **7.2 (1.9-26.6)** | 2.0 (0.6-6.6) |
|  | Remote or very remote | 1 | 1 |  | 1 | 1 |
| Jurisdiction | Northern Territory/ South Australia | 1 | 1 |  |  |  |
|  | New South Wales | 1.1 (0.1-8.7) | **2.4 (1.0-5.8)** |  |  |  |
|  | Western Australia | 1.5 (0.7-3.5) | 0.7 (0.2-2.0) |  |  |  |
| Birth period | 2002-2009 | 1 | 1 |  |  |  |
|  | 2010-2017 | 0.8 (0.4-1.7) | 0.7 (0.3-1.5) |  |  |  |
| Valvular surgery prior to 20 weeks gestation | | NA | 1.4 (0.6-3.2) |  |  |  |
| First antenatal visit before 20 weeks gestation | | 0.8 (0.3-1.8) | 1.1 (0.5-2.5) |  |  |  |
| Comorbidities and prior complications | Complicated previous pregnancy | **4.3 (1.6-11.3)** | **2.6 (1.1-6.6)** |  | 2.4 (0.8-7.5) | 1.6 (0.4-6.0) |
|  | Anticoagulants | **3.8 (1.1-13.4)** | 1.3 (0.3-4.8) |  | 1.1 (0.3-4.4) | 1.9 (0.3-11.7) |
|  | Cardiometabolic disease^†^ | **5.1 (2.3-10.8)** | 2.3 (0.9-5.7) |  | **3.3 (1.3-8.4)** | 1.4 (0.3-5.5) |
|  | Anaemia | **4.6 (2.2-9.7)** | 1.6 (0.7-3.5) |  | **2.6 (1.0-6.9)** | 1.3 (0.4-4.0) |
|  | Behavioural factors^‡^ | **2.7 (1.3-5.8)** | 1.7 (0.8-3.7) |  | 1.2 (0.5-3.0) | 1.3 (0.4-4.2) |

* Parity, plurality, inter-pregnancy interval, birth setting and birth attendant variables were excluded from this analysis due to large amount of missing information.

† Cardiometabolic disease – IHD, COPD, CKD, diabetes, hypertension.

‡ Behavioural factors – chronic alcohol use, mental health-related, smoking

§ Multivariate analysis shows the data presented graphically in Figure 2B.

## Supplement 5. Baseline demographic features of women with RHD who became pregnant during 2010-2017, stratified by RHD complication status at 20 weeks of index pregnancy during the study period.

|  | **RHD status at first recorded pregnancy***  (n, column %) | |
| --- | --- | --- |
|  | **Uncomplicated** **RHD** (n=271) | **Complicated RHD**  (n=41) |
| **Maternal age in years** |  |  |
| <20 years | 48 (17.7) | 6 (14.6) |
| 20-29 years | 170 (62.7) | 23 (56.1) |
| 30-44 years | 53 (19.6) | 12 (29.3) |
| **Maternal population group** |  |  |
| First Nations | 259 (95.6) | 33 (80.5) |
| Born in low/middle income country | 8 (3.0) | 7 (17.1) |
| Other Australian | <5 | <5 |
| **Year of RHD diagnosis** |  |  |
| 2002-2009 | 174 (64.2) | 25 (61.0) |
| 2010-2017 | 97 (32.8) | 16 (39.0) |
| **Jurisdiction of residence** |  |  |
| Northern Territory / South Australia | 186 (68.6) | 21 (51.2) |
| Western Australia | 71 (26.2) | 9 (22.0) |
| New South Wales | 14 (5.2) | 11 (26.8) |
| **Geographical remoteness** |  | |
| Major cities/ inner regional | 14 (5.2) | 13 (31.7) |
| Outer regional | <5 | <5 |
| Remote/ very remote | 253 (93.4) | 27 (65.9) |
| **Index of Socioeconomic disadvantage** |  | |
| More/most disadvantaged | 253 (93.4) | 34 (82.9) |
| Median disadvantage | 9 (3.3) | <5 |
| Less/least disadvantaged | 6 (2.2) | <5 |
| Unknown | <5 | 0 |

* subset of pregnancies shown in Figure 2A, first pregnancy per woman.

### Data from Figure 2C

Risk of RHD-associated MACE, estimated with pregnancy as time varying predictor.

|  | **Complicated** | **Uncomplicated** |
| --- | --- | --- |
| Age over 30 years | 1.82 (0.93-3.55) | 1.06 (0.49-2.29) |
| Pregnancy | 3.05 (1.55-5.99) | 5.11 (2.73-9.55) |
